# Chemotherapeutic and targeted agents can modulate the tumor microenvironment and increase the efficacy of immune checkpoint blockades

**DOI:** 10.1186/s12943-021-01317-7

**Published:** 2021-02-04

**Authors:** Jun-Yan Li, Yu-Pei Chen, Ying-Qin Li, Na Liu, Jun Ma

**Affiliations:** Department of Radiation Oncology, State Key Laboratory of Oncology in South China, Collaborative Innovation Center for Cancer Medicine, Guangdong Key Laboratory of Nasopharyngeal Carcinoma Diagnosis and Therapy, Sun Yat-sen University Cancer Center, 651 Dongfeng Road East, Guangzhou, 510060 People’s Republic of China

**Keywords:** antitumor immune response, chemotherapy, combination therapy, immune checkpoint blockade, targeted therapy, tumor microenvironment

## Abstract

The development of immune checkpoint blockade (ICB)-based immunotherapy has dramatically changed methods of cancer treatment. This approach triggers a durable treatment response and prolongs patients' survival; however, not all patients can benefit. Accumulating evidence demonstrated that the efficacy of ICB is dependent on a robust antitumor immune response that is usually damaged in most tumors. Conventional chemotherapy and targeted therapy promote the antitumor immune response by increasing the immunogenicity of tumor cells, improving CD8+ T cell infiltration, or inhibiting immunosuppressive cells in the tumor microenvironment. Such immunomodulation provides a convincing rationale for the combination therapy of chemotherapeutics and ICBs, and both preclinical and clinical investigations have shown encouraging results. However, the optimal drug combinations, doses, timing, and sequence of administration, all of which affect the immunomodulatory effect of chemotherapeutics, as well as the benefit of combination therapy, are not yet determined. Future studies should focus on these issues and help to develop the optimal combination regimen for each cancer.

## Introduction

Immune checkpoint blockade (ICB)-based immunotherapy has resulted in a revolutionary shift in cancer treatment. Distinguished from conventional chemotherapy and radiotherapy, which suppress tumors by directly killing malignant cells, ICBs rescue the antitumor activity of T cells through targeted blockade of checkpoints, such as cytotoxic T lymphocyte-associated protein 4 (CTLA-4), programmed cell death 1 (PD-1), and its ligand PD-L1 (also known as CD274), and are superior in establishing immune memory and preventing recurrence [1]. In the past decade, the clinical uses of ICBs have shown promising results in the treatment of many different kinds of malignancies [2–4]. To date, several distinct ICBs, including (1) the CTLA-4 antibody ipilimumab (Yervoy); (2) the PD-1 inhibitors: Cemiplimab (Libtayo), nivolumab (Opdivo), and pembrolizumab (Keytruda); and (3) the PD-L1 blockers: Atezolizumab (Tecentriq), avelumab (Bavencio), and durvalumab (Imfinzi), have been approved to treat a variety of advanced cancers, including melanoma, non-small cell lung cancer (NSCLC), hepatocellular carcinoma (HCC), head and neck squamous cell carcinoma (HNSCC), and urothelial carcinoma. Furthermore, these and several other ICBs are under clinical test and are expected to expand the panel of oncological indications.

Despite their increasing varieties and indications, ICBs have been demonstrated to induce an effective and durable antitumor immune response only in a small subset of patients. The response rates to ICBs used as stand-alone therapeutic interventions in unselected patients are mostly less than 30% in a variety of tumor types [5], which is unsatisfactory. Further improving the antitumor efficacy of ICB-based immunotherapy has become one of the main challenges in clinical oncology.

The clinical efficacy of ICBs depends on the pre-existing antitumor immunity. Immune checkpoints are the negative regulators of antitumor immunity. PD-1 and CTLA-4 are expressed on T cells during priming and activation, while in the tumor microenvironment (TME), local interferon-gamma (IFNγ), mainly derived from effector lymphocytes, induces the expression of PD-L1 on cancer cells and intra-tumoral immune cells. Accumulating evidence suggests that tumors infiltrated by CD8+ T cells that can recognize and kill cancer cells are more likely to respond to ICB treatment [6].

Historically, conventional chemotherapy was considered as immunosuppressive because it broadly affects immune cells, in addition to tumor cells, resulting in myelosuppression and leukopenia. However, recent studies have demonstrated that chemotherapy can activate an endogenous antitumor immune response, which partly contributes to their therapeutic effects. Similar immunomodulatory effects were also observed for targeted agents, such as tyrosine kinase inhibitor (TKI), which originally inhibited the proliferation of neoplastic cells with cancer-specific alterations, likely because of the shared signaling pathways between cancer and immune cells. Following these findings, both conventional chemotherapeutics and targeted agents have been suggested to be combined with ICBs to enhance antitumor efficacy. Previous treatment success of the combination therapies confirmed their synergistic effect [7–10] and encouraged further investigations. Given the clinical momentum in combining these two classes of therapies, it is crucial to understand the actions of chemotherapeutics on the antitumor immune response. Here, we summarize the discovered immunomodulatory chemotherapeutics and targeted agents, discuss how they modify antitumor immunity, and review the possibility of combining these medications with ICBs.

## Antitumor CD8+ T-cell immunity – the basis for ICB treatment efficacy

CD8+ cytotoxic T lymphocytes (CTLs)-mediated antitumor immunity is the backbone of immune elimination of cancer, as well as the basis for the effectiveness of the ICB. This multistep event is also termed as the cancer-immunity cycle. It starts with the release of neoantigens created by oncogenesis (step 1). Next, antigen-presenting cells (APCs), such as dendritic cells (DCs), will capture these neoantigens and migrate to the draining lymph nodes, where they present the processed peptide to naïve T cells (step 2); leading to the priming and activation of tumor-specific T cells (step 3). During activation, T cells also acquire a chemotactic ability toward the tumor via their expression of C-X-C motif chemokine receptor 3 (CXCR3), a chemokine receptor that can bind to cancer-derived ligands (such as C-X-C motif chemokine ligand, CXCL9, CXCL10, and CXCL11). Then, under the chemokine-receptor interaction, the activated T cells traffic to (step 4) and infiltrate into the tumor bed (step 5), where they specifically recognize (step 6) and eventually kill their target cancer cells (step 7) [11].

**Fig. 1:**
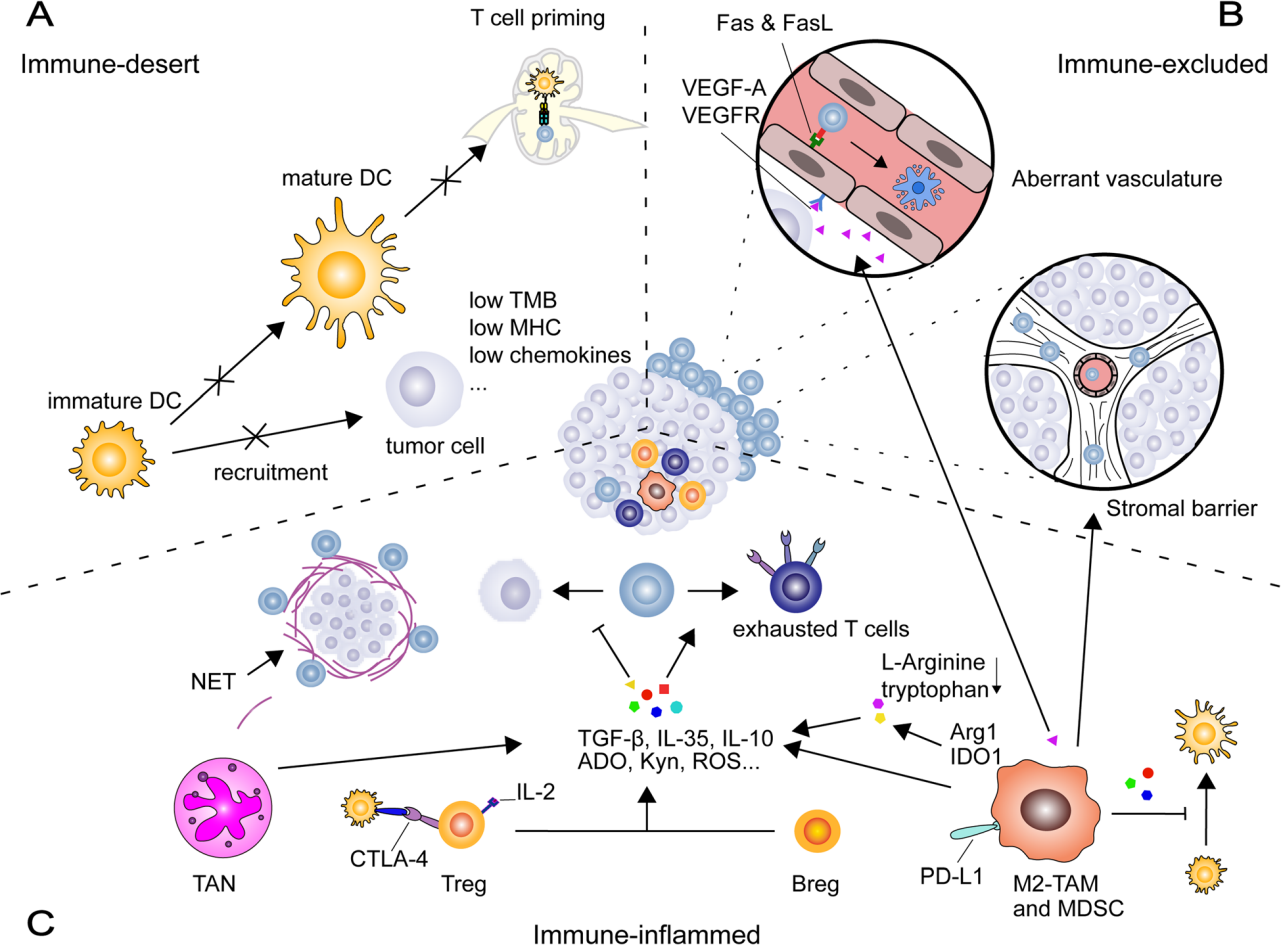
Failure of cancer-immunity cycles in three different tumor-immune landscapes. **a** In the immune-desert tumor, the generation of tumor-specific CD8+ T cells is impaired because of low immunogenicity. **b** In the immune-excluded tumor, CTL infiltration is impaired because the arriving tumor-specific CD8+ T cells are shut out by the aberrant vasculature and stromal barriers. **c** In the immune-inflamed tumor, the immunosuppressive cells directly impair the cytotoxicity of tumor-specific CD8+ T cells and indirectly inhibit T cell activation by suppressing DCs. Arg1: argase1; Breg: regulatory B cell, CTLA-4: cytotoxic T lymphocyte-associated protein 4, DCs: dendritic cells, IDO1: indoleamine 2,3-dioxygenase 1, IL: interleukin, MHC: major histocompatibility complex, NETs: neutrophil extracellular traps, PD-1/PD-L1: programmed cell death 1 and its ligand 1, TAM: tumor-associated macrophage, TAN: tumor-associated neutrophils, TGFβ: transform grow factor-β, TMB: tumor mutation burden, Treg: regulatory T cell, VEGF-A & VEGFR: vascular endothelial growth factor A and its receptor

Immune escape from ICB-based immunotherapy has been attributed to failures in the steps of the cancer-immunity cycle, which varies in different tumor types [12]. Various cellular and humoral factors in the TME drive or reduce anticancer immunity to account for these failures. They constitute different tumor immune landscapes and have been demonstrated to be associated with the tumor response to ICBs (Fig. 1).

Tumor immunogenicity plays a central role in initiating the antitumor immune response [13]. This depends on two key factors: (1) tumor antigenicity, i.e., the tumor neoantigens which can be recognized as non-self compounds; and (2) immune adjuvanticity, i.e., the inflammatory signals that promote the recruitment, maturation, and antigen presentation of immune cells such as DCs. In other words, the tumor immunogenicity favors the generation and recruitment of tumor-specific CTLs, avoiding the immune-desert phenotype which indicates a non-response to ICB treatment. Consistent with these notions, highly-mutated malignancies with an abundance of neoantigens, such as melanoma, NSCLC, and HNSCC, are more sensitive to ICB treatment [14, 15]. Patients with colorectal cancer (CRC) with high microsatellite instability or defects in the mismatch repair system are likely to have improved tumor control after treatment with PD-1/CTLA-4 inhibitors [16, 17].

Another prerequisite for a successful ICB response is the infiltration of activated tumor-specific CTLs. High intra-tumoral CTL levels are recognized as a predictor of improved response and treatment outcome of ICB therapy, while immune-excluded tumors, with T cells present at the invasive margin, usually respond less to ICBs. Accumulating evidence suggests that the desmoplastic stroma and disorganized tumor vasculature of the TME are the main reasons for the immune-excluded phenotype. In line with this notion, single-agent ipilimumab has been demonstrated to be ineffective to treat advanced pancreatic ductal adenocarcinoma (PDAC), a tumor characterized by fibrotic stroma [18]. Furthermore, vascular endothelial growth factor A (VEGF-A), which promotes the tumor vasculature, is associated with treatment resistance to anti-CTLA-4 antibodies in patients with melanoma [19], likely because it impairs endothelial-T cell adhesion and subsequent T-cell infiltration by reducing levels of intercellular adhesion molecule–1 (ICAM-1), as well as vascular cell adhesion molecule–1 (VCAM-1) [20], and triggers CD8+ T cell apoptosis by inducing FASL on endothelial cells [21].

Immunosuppressive cells are the most important suppressors for the antitumor immune response. Previous studies have confirmed the immunosuppressive roles of regulatory T cells and B cells (Tregs and Bregs), anti-inflammatory tumor-associated macrophages (M2-TAMs), tumor-associated neutrophils (TANs), and myeloid-derived suppressor cells (MDSCs) in various kinds of cancers. These populations are selectively accumulated and activated in tumor sites by either cancer-cell secreted chemokines or chronic inflammatory signals. Cellular and humoral factors have been exploited to suppress CTL-mediated antitumor response, including (1) immunosuppressive cytokines, such as interleukin (IL)-10, IL-35, and transforming growth factor-beta (TGF-β), which inhibit DC maturation and antigen presentation; T-cell activation; and the priming and cytotoxicity of CTLs; (2) immunosuppressive metabolites, including adenosine, kynurenine, reactive oxygen species, lactic acid, and nitric oxide are generally derived from MDSCs, M2-TAMs, Tregs, and tumor-associated neutrophils. These molecules not only directly impaired CTL antitumor capacity by decreasing effective molecules like IFNγ and perforin, and upregulating co-inhibitory receptors such as PD-1 and TIM3, but also positively increased the recruitment of suppressor cells, thereby exacerbating immunosuppression; (3) immunosuppressive ligands: M2-TAMs, TANs, Bregs, and MDSCs usually have high levels of surface inhibitors, such as PD-L1, while Tregs can also inactivate DCs via the expression of CTLA-4, which binds to CD80/CD86 on DCs and transduces suppressive signals to interrupt DC activation [22]; (4) consumption of key nutrients and grow factors: Tregs competitively consume IL-2, while M2-TAMs and MDSCs can reduce local L-cysteine, L-arginine, and tryptophan, thereby restricting the activation and proliferation of CTLs; (5) vascularization and stroma promotion: both M2-TAMs and MDSCs are important sources of VEGF-A and they prevent CTL infiltration by promoting the proliferation of cancer-associated fibroblasts [23, 24]; and (6) cancer cell protection: Neutrophil extracellular traps wrap and coat tumor cells and thus protect them from CTL-mediated killing [25].

In addition to immunosuppression, these populations, especially M2-TAMs, play important roles in tumorigenesis. Tissue chronic inflammation mediated by macrophages was thought to be mutagenic and growth-promoting. With the expression of pro-tumoral cytokines, such as EGF and CCL18, M2-TAMs, MDSCs, and TANs directly activate the migration of cancer cells [26–28]. M2-TAM and MDSCs induce the rapid generation of immature vascular networks providing the nutrients and oxygen for tumor proliferation [29]. M2-TAMs can also enhance tumor invasion directly [30], and induce the entrance of tumor cells into circulation [31]. In the metastatic tumor microenvironment, M2 macrophage assist tumor cell extravasation from blood vessels [32] and supported the seeding, survival, and prospering of tumor cells through the formation of a nurturing niche [33, 34]. Consistent with their tumor promotion and immune suppression effects, accumulating evidence suggests that the high accumulation of Tregs, Bregs, M2-TAMs, TAN, or MDSCs is associated with a poor response to ICB treatment in a variety of cancers [35, 36].

In contrast to suppressive immune populations, CTL-promoting cells are also present in the TME and are usually related to a better response to ICB therapy. Natural killer (NK) cells and gamma/delta T cells (γ/δ T cells) are the important lymphocytes in the innate immune system. In an MHC-independent cytolytic manner, these two populations can effectively deplete tumor cells with antigen-presentation deficiency, and thus serve as allies of antitumor CTLs. Besides, previous studies have found that both NK and γ/δ T cells can promote the generation of CTLs. NK cells can recruit conventional DCs to the tumor bed via chemo-attractants, such as XCL1, CCL5, and Fms-related tyrosine kinase 3 ligand (FLT3LG) [37, 38], while γ/δ T cells can directly act as professional APCs [39]. B cells are also key professional APCs in cancer. Recent studies have demonstrated that they participate in the formation of tertiary lymphoid structures (TLS), allowing the generation of tumor-specific CTLs, and eventually driving the tumor response to ICB treatments [40–42].

## Chemotherapeutics promote antitumor immunity (Fig. 2)

Chemotherapeutics originally direct inhibited or killed malignant cells to achieve their therapeutic effects. Recently, some frontline drugs have been found to additionally promote antitumor immunity by increasing tumor immunogenicity, improving T cell infiltration, or depleting the immunosuppressive populations. Therefore, it is reasonable to hypothesize that chemotherapeutics specifically remove the constraints of the antitumor immune response in different TMEs, making them the first-line option for these tumors. The immunomodulatory effects of some popular chemotherapeutics are summarized in Table 1. Some of them have been well-reviewed previously [85]; therefore, the current review mainly focuses on recent findings and their role in combination therapy with ICBs.

**Fig. 2:**
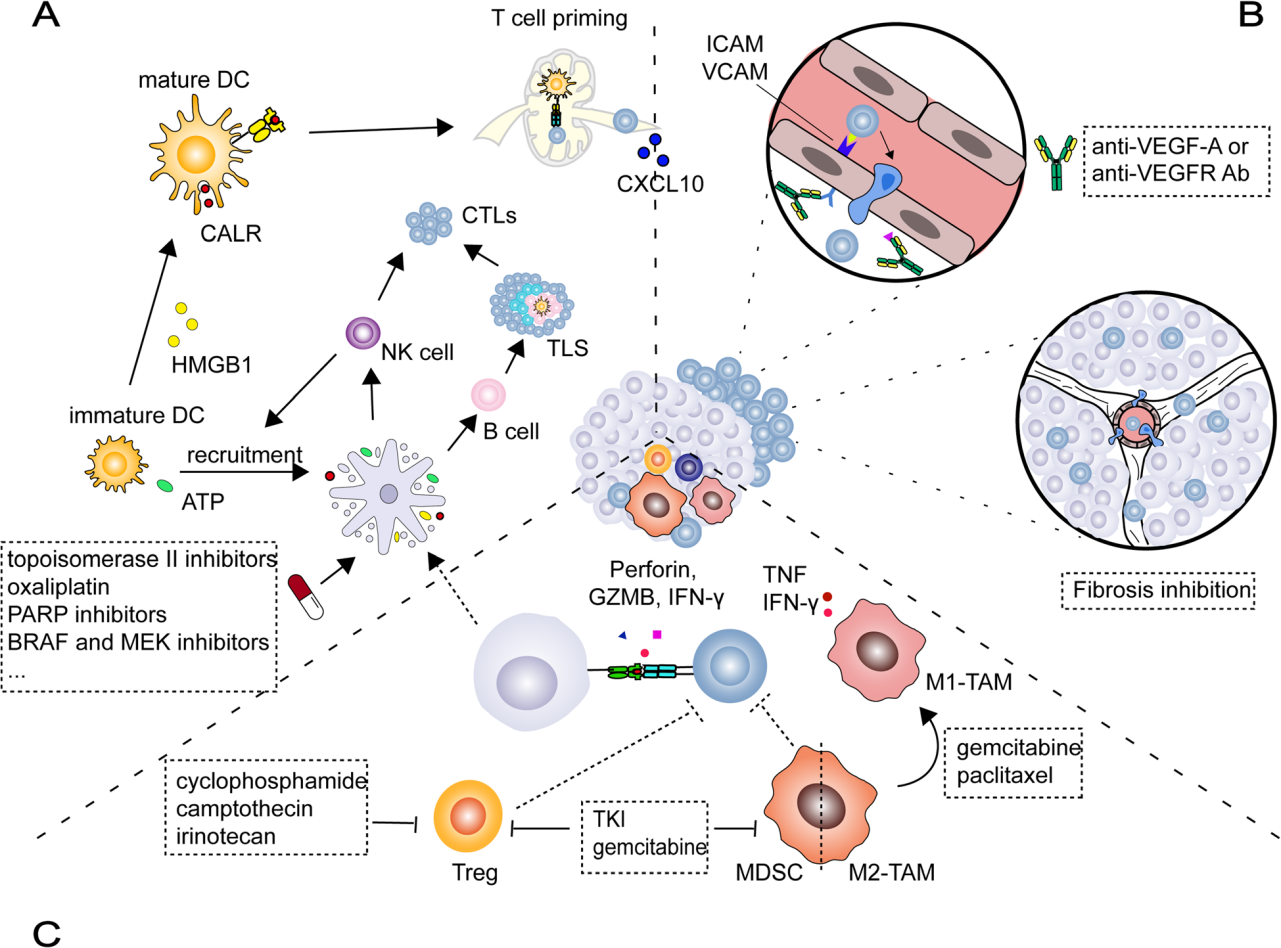
Chemotherapeutics drive cancer-immunity cycles. Chemotherapeutics induce immunogenic cell death (**a**), promote CD8+ T cell infiltration (**b**), and inhibit immunosuppressive cells (**c**). ATP: adenosine-triphosphate, BRAF: B-Raf proto-oncogene, serine/threonine kinase, CTL: Cytotoxic T lymphocytes, CXCL10: C-X-C motif chemokine ligand 10, DC: dendritic cells, HMGB1: high-mobility group box1, ICAM-1: intercellular adhesion molecule, IFN-γ: interferon-gamma, MDSC: myeloid-derived suppressor cell, NK cells: natural killer cells, PARP: Poly (ADP-ribose) polymerase, TAM: tumor-associated macrophage, TKI: tyrosine kinase inhibitor, TLS: Tertiary lymphoid structures, TNF: tumor necrosis factor, Treg: regulatory T cell, VCAM: vascular cell adhesion molecule

**Table 1:** Immunological Effects of Conventional Antitumor Agents

Agent	Setting	Effect	Notes	Reference
**Chemotherapeutic agents**				
Anthracyclines	various murine cancers	induced ICD	by activating type I IFN pathway and releasing HMGB1	Sistigu et al [43] Apetoh et al [44]
	transplantable murine colon cancer	induced ICD	by increasing the exposure of calreticulin	Obeid et al [45]
Cisplatin	human HCC	increased NK cell cytotoxicity	by upregulating UL16-binding protein 2, a natural-killer group 2 member D ligand	Shi et al [46]
	human ovarian cancer and transplantable murine ovarian cancer	promoted NK cell infiltration and T cell oligoclonal expansion	increased tumor immunogenicity	Jiménez-Sánchez et al [47]
Cyclophosphamide	transplantable murine fibrosarcoma	reduced Tregs and then enhanced priming of tumor-specific CD8+ T cells	specifically eradicated the proliferating and suppressive CCR2+ Tregs	Loyher et al [48]
	human metastatic CRC	reduced the number of peripheral B cells and Tregs	a low dose of Cyclophosphamide expanded and activated tumor-specific T cells	Scurr et al [49]
Camptothecin	transplantable murine Lewis lung cancer and colon adenocarcinoma	reduced the generation and activation of Tregs	inhibited the expression of FOXP3	Hibino et al [50]
Docetaxel	human NSCLC	promoted CD8+ T-cell recruitment	increased the release of HMGB1 and CXCL11	Gao et al [51]
Doxorubicin, cyclophosphamide, and paclitaxel combination regimen	human breast cancer	increased the ICOSL+ B cell subset and promoted anti-tumor T cell immunity	Complement signals initiated by immunogenic cell death shape B cell phenotypes	Lu et al [52]
5-Fluorouracil	transplantable murine CRC	favored MDSC differentiation, relieving immunosuppression	in the context of FOLFIRI	Kanterman et al [53]
Gemcitabine	human PDAC	increased antigen presentation and chemokines	increased the TGFβ-associated signals and cancer-associated fibroblasts	Principe et al [54]
	human PDAC	reprogrammed TAM to a pro-inflammatory phenotype	gemcitabine changed the negative prognosis effect of TAM to positive	Di Caro et al [55]
Irinotecan	human CRC	reduced peripheral Tregs	in the context of FOLFIRI	Roselli et al [56]
Oxaliplatin	transplantable murine colon cancer	induced ICD	by increasing the release of ATP	Ghiringhelli et al [57]
Paclitaxel	transplantable murine ovarian cancer	induced ICD	by increasing exposure of calreticulin	Garg et al [58]
	transplantable murine breast cancer, melanoma, and PDAC	reprogramed TAM to a pro-inflammatory phenotype	in a TLR4 dependent manner	Wanderley et al [59] Cullis et al [60]
Pemetrexed	transplantable murine colon cancer	induced ICD	also augmented T cell mitochondrial function and enhances activation	Schaer et al [61]
Teniposide	transplantable murine colon cancer	induced ICD	also enhanced the expression of tumor antigen presentation machinery molecules	Wang et al [62]
**Targeted agents**				
Abemaciclib	murine ovarian cancer	augmented T cell and B cell infiltration	by upregulating the chemokines CXCL10 and CXCL13	Zhang et al [63]
Bevacizumab	human metastatic RCC	augmented T-cell infiltration	through vessel normalization, endothelial cell activation, and T cell recruitment	Wallin et al [64]
BRAF inhibitor (PLX4720)	transplantable murine melanoma	induced pyroptosis	in combination with MEK inhibitor	Erkes et al [65]
Bortezomib	human glioblastoma, synovial sarcoma, and pancreatic adenocarcinoma	enhanced NK cell cytotoxicity	by the upregulation of cell surface NK ligands MHC class I chain-related protein A and B	Luna et al [66]
Cetuximab	transplantable murine colorectal cancer	induced ICD	*BRAF*^V600E^ mutation impeded the induction of ICD	Pozzi et al [67]
Capmatinib	murine melanoma	reduced reactive neutrophil recruitment to tumors and lymph nodes	activated T cell antitumor immunity and enhanced the efficacy of cancer immunotherapies	Glodde et al [68]
Crizotinib	murine non-small-cell lung carcinoma	induced ICD	Crizotinib plus cisplatin increased the expression of PD-1 and PD-L1 in tumors	Liu et al [69]
MEK inhibitor (cobimetinib)	transplantable murine colon cancer	increased the number of intra-tumoral CD8+ T cells	can also potentiate anti-tumor T cells by impairing TCR-driven apoptosis	Ebert et al [70]
MEK inhibitor (selumetinib)	transplantable murine colon cancer	reduced intra-tumoral immunosuppressive myeloid cells	pre-treatment, but not concurrent treatment, with selumetinib enhanced the anti-tumor activity of anti-CTLA-4	Poon et al [71]
PARP inhibitor (olaparib)	murine *BRCA*-deficient TNBC	induced CD8+ T cell recruitment	by activating cGAS/STING pathway	Pantelidou et al [72]
	murine NSCLC	induced CD8+ T cell recruitment	increased PD-L1 expression on tumor cells	Sen et al [73]
PARP inhibitor (talazoparib)	human ovarian cancer cell, transplantable murine ovarian cancer	induced CD8+ T cell infiltration	regardless of BRCA-mutant status	Shen et al [74]
PI3K-γ inhibitor (IPI-549)	murine pancreatic cancer and melanoma	reduced the number of intra-tumoral immunosuppressive plasma cells	also reduced the number of myeloid cells	Zhang et al [75]
TKI (Dasatinib)	transplantable murine cancers	reduced intra-tumoral MDSC and Tregs	enhanced the activity of CTLs	Lowe et al [76] Hekim et al [77]
TKI (Cabozantinib)	murine prostate cancer	increased neutrophil infiltration and activated innate antitumor immunity	through the release of neutrophil chemotactic factors from tumor cells, including CXCL12 and HMGB1	Patnaik et al [78]
TKI (Sunitinib)	human metastatic RCC	reduced the number of peripheral Tregs and MDSCs	increased the number of IFNγ+ T cells	Finke et al [79] Ko et al [80]
	murine HCC	decreased the number of peripheral and tumor-infiltrating Tregs, and reduced IL-10 and TGF-β	recovered antitumor activity of CD8 T cell; favored PD-1 blockade	Liu et al [81], Li et a l[82]
VEGFR2 targeting antibody	human advanced gastric cancer	increased CD8+ T cell infiltration	also decreased Tregs	Tada et al [83]
	murine breast cancer	increased CD8+ T cell infiltration	also increased DCs	Allen et al [84]

ATP: adenosine-triphosphate; BRAF: B-Raf proto-oncogene, serine/threonine kinase; BRCA: breast cancer susceptibility gene; CCR2: chemokine (C-C motif) receptor 2; cGAS/STING: cyclic GMP–AMP synthase/stimulators of interferon gene; CXCL12: C-X-C Motif Chemokine Ligand 11; CRC: colon rectal cancer; CTL: cytotoxic T lymphocyte; CTLA-4: cytotoxic T lymphocyte-associated protein 4; DC: dendritic cell; FOLFIRI, folinic acid, 5-fluorouracil; FOXP3: Forkhead Box P3; HCC: hepatocellular carcinoma; HMGB1: high-mobility group box1; ICD: immunogenic cell death; IFNγ: interferon-γ; IL: interleukin; MDSC: myeloid-derived suppressor cell; NSCLC: non-small cell lung cancer; PD-1/PD-L1: programmed cell death 1 and its ligand 1; PDAC: pancreatic ductal adenocarcinoma; RCC: renal cell cancer; TAM: tumor-associated macrophage; TCR: T cell receptor; TGFβ: transform grow factor-β; TKI: tyrosine kinase inhibitors; TLR4: toll-like receptor 4; TNBC: triple-negative breast cancer; Tregs: regulatory CD4+ T cells; VEGFR: vascular endothelial growth factor receptor

### Chemotherapeutic and targeted agents activate antitumor CD8+ T cell immunity

Decreased immunogenicity is one of the most important characteristics of malignancies, leading to immune ignorance. Besides, in most cases, chemotherapeutics induce apoptosis and necrosis of cancer cells, which is incapable of inducing adaptive immunity. By contrast, immunogenic cell death (ICD) is characterized by its potential to increase tumor immunogenicity and thus establish long-lasting antitumor immunity. To date, several kinds of chemotherapeutics that have been commonly used in clinical practice were found to mediate their antineoplastic activity by inducing ICD of malignant cells [86].

Anthracyclines, oxaliplatin, and paclitaxel are well-recognized as ICD inducers. Preclinical and clinical histological observations have demonstrated that these agents significantly increase the abundance of intra-tumoral CD8+ T cells, which favors their antineoplastic efficacy and is associated with better patient outcomes [85, 87]. The mechanisms through which they induce ICD have been determined [13]. Traditionally, several processes, such as the unfolded protein response, autophagy, inflammasome signaling, Toll-like receptor 3 (TLR3) signaling, and type 1 interferon response are related to ICD. During these processes, damage-associated molecular patterns (DAMPs) including adenosine triphosphate (ATP), the surface-exposure of calreticulin, high-mobility group box1 (HMGB1) promoted DC-mediated CTL activation; and chemokines such as CXCL10 enhanced CTL recruitment. Moreover, recent studies have found that ICD-inducing drugs can also modulate antitumor CTL immunity through tumor-infiltrating NK cells and B cells. In human ovarian cancer, platinum and taxane chemotherapy significantly increase NK cell infiltration and local T cell oligoclonal expansion [47]. While in human breast cancer, the neoadjuvant doxorubicin, cyclophosphamide, and paclitaxel combination regimen switched the tumor-infiltrating B cells to a new ICOSL+ phenotype. These newly emerging B cells are involved in the formation of the TLS, and significantly increased the numbers and the cytotoxicity of tumor-specific CD8+T cells [52] (Fig. 1a).

Given their ability to activate the antitumor CTL response, ICD-inducing chemotherapeutics are believed to combine with and enhance the therapeutic efficacy of ICBs. Doxorubicin plus PD-1 or PD-L1 antibodies showed a significantly improved antitumor effect in various murine cancers, such as melanoma and breast cancer [88, 89]. In human metastatic triple-negative breast cancer (TNBC), short-term doxorubicin induction sensitized the tumor to PD-1 blockade [90]. Similarly, oxaliplatin was demonstrated to boost the efficacy of anti-PD-L1 therapy in murine colorectal cancer [91]. Paclitaxel and ICB combination therapy elicited a superior tumor-suppression effect in nonimmunogenic squamous NSCLC [8].

In addition to anthracyclines, teniposide, another topoisomerase II inhibitor, was reported recently to induce ICD; however, it acts via a different mechanism to that of anthracyclines. Topoisomerase II inhibitors induced the proliferation-arrest or demise of neoplastic cells by increasing DNA double-strand breaks [92]. It has been suggested that damaged DNA fragments in the nucleus could be actively exported to the cytoplasm, possibly to prevent misincorporation into genomic DNA, and trigger the innate immune response mediated by the cGAS-STING (cyclic GMP–AMP synthase/stimulators of interferon gene) pathway [93–95]. In line with this notion, teniposide activated the tumor-cell intrinsic type-I interferon (IFN) response and upregulated features of ICD. Besides, teniposide strengthened the tumor cell antigen presentation machinery, which augmented T-cell recognition. Consequently, in rodent colon cancer, teniposide induced robust antitumor CD8+ T-cell immunity and remarkable tumor suppression. Vaccination with teniposide-treated dead tumor cells effectively prevented tumor redevelopment. Furthermore, the administration of teniposide successfully reversed the insensitivity to the PD-1 inhibitor of *KRAS* mutant CT26 colon cancer [62]. Despite its positive immunomodulatory effect in murine tumors, whether teniposide acts as an ICD inducer in human cancers remains elusive.

Poly (ADP-ribose) polymerase inhibitors (PARPi), including olaparib and niraparib, inhibit DNA repair in homologous-recombination-deficient malignant cells, leading to synthetic lethality [96]. Such retention and accumulation of DNA damage can activate the cGAS-STING pathway and the subsequent type-I IFN response, as mentioned above. In line with this notion, the administration of olaparib to murine *BRCA* (encoding breast cancer type 1 susceptibility protein) -deficient TNBCs increased the CD8+ T cell abundance and activated antitumor immunity [72]. Despite PARPis generally eliciting antitumor efficacy in *BRCA*-mutant cancers, clinical investigations have demonstrated the unexpected treatment benefits of niraparib in patients with BRCA-proficient ovarian cancer [97, 98]. A recent preclinical study found that in ovarian cancer, PARPi triggered the STING-dependent immunogenic response, regardless of DNA repair deficiency [74]. A similar observation was also found in small cell lung cancer (SCLC) [73]. In addition to the increased intra-tumoral CTLs, PARPi could upregulate PD-L1 expression in malignant cells in breast cancer, SCLC, and ovarian cancer, regardless of the *BRCA* mutation status. Such increasing CTL abundance and intra-tumoral PD-L1 level potentiate the combined therapy of PARPi and ICBs [99]. As expected, a combination of niraparib plus pembrolizumab therapy showed promising synergistic antitumor activity in patients with TNBC or ovarian cancer [100, 101], despite the best treatment efficacy still being observed in patients with *BRCA*-mutant [102]. Although the combination of a PARPi and an ICB (olaparib plus durvalumab) did not show satisfactory therapeutic efficacy in SCLC, it is worth noting that the addition of olaparib might be capable of reversing the ICB resistance of SCLC, because some tumors that progressed in previous ICB treatment maintained stable disease under the combined regimen [103].

Pyroptosis is a new pattern of cell death, which is mediated by gasdermin (GSDM) proteins. GSDMs, mainly GSDMD and GSDME, are activated after the cleavage of their autoinhibitory N domains by caspases. They translocate to, and form pores on, the cytomembrane, resulting in cell swelling, membrane rupture, and the release of cytosolic contents including DAMPs, such as HMGB1 and ATP [104, 105]. Some conventional chemotherapeutic agents, like cisplatin and etoposide, can induce pyroptosis. However, *GSDME* is silenced in most cancer cells, but is expressed in many normal cells, including lymphocytes; therefore, these medications were traditionally supposed to impair, rather than promote, antitumor immunity [106, 107]. Intriguingly, a recent study showed that GSDME-mediated pyroptosis acts as a form of ICD and effectively activated antitumor CD8+ T-cell immunity in murine melanoma [108]. The combination of B-Raf proto-oncogene, serine/threonine kinase (BRAF) and MAPK/ERK kinase (MEK) inhibitors, the frontline care for *BRAF*^V600E^-mutant melanoma, was found to induce the pyroptosis of melanoma cells by blocking extracellular regulated kinase (ERK)1/2 signaling and subsequently activating the GSDME cleaver, caspase-3. These dual inhibitions significantly increased the intra-tumoral abundance of DCs, as well as CTLs, contributing to durable tumor regression [65]. Besides, BRAF inhibition alone has been demonstrated to increase CD8+ T cells, while MEK inhibitors potentiated anti-tumor T cells by preventing T-cell receptor (TCR)-driven apoptosis [70]. The potential of BRAF and MEK inhibitors to synergize the effects of anti-PD-1 antibodies has been observed in mouse melanoma [109]. In human *BRAF*^V600E^-mutant melanoma, such a triplet therapy facilitated a remarkable antitumor response and prolonged the progression-free survival of patients [110, 111]. Similar to BRAF and MEK inhibitors, crizotinib, which is used to treat NSCLC carrying activated anaplastic lymphoma kinase (ALK) and ROS proto-oncogene 1, receptor tyrosine kinase (ROS1), favored the ICD, likely because it triggered the pyroptosis of lung cancer cells in which GSDME is expressed ubiquitously [112]. The administration of crizotinib increased CTL accumulation in murine NSCLC and remarkably sensitized the tumor to PD-1 blockade [69].

### Chemotherapeutic and targeted agents enhance CD8+ T-cell infiltration

In addition to promoting the generation of CTLs, chemotherapeutics can enhance their entry into the tumor center. Antiangiogenic molecules that target the VEGF/VEGFR axis are expected to starve the tumor, thus suppressing tumor progression and improving patient survival. However, recent research has found that, instead of simply starving tumor cells to death, antiangiogenic agents also promote immune attack by markedly increasing the infiltration of tumor-specific CTLs after normalizing the immature vessels (Fig. 1b).

In a variety of rodent and human malignancies, both anti-VEGF-A and anti-VEGFR2 agents have been demonstrated to increase T cell infiltration [64, 113], and dual inhibition of the VEGF/VEGFR axis, and other antiangiogenic factors, such as angiopoietin-2 (ANGPT2) and prostaglandin E2 (PGE2), further improved this increment [21, 114]. After infiltration, CTLs recognize the tumor cells and secrete many cytotoxic cytokines, such as IFN-γ, resulting in the induction of PD-L1 expression by tumor cells. Thus, it provides a convincing rationale for the development of the combination of antiangiogenic therapy and immunotherapy. As expected, different combinatorial treatments of antiangiogenic agents and ICBs have shown a higher synergistic effect in tumor control compared with that achieved by monotherapy in various rodent cancers, including breast cancer, pancreatic neuroendocrine tumor, and HCC [84, 113]. Histological examinations showed that the combination of ICB and antiangiogenic agents further promoted vessel normalization, even toward high endothelial venules [84]. Such normalized vessels permitted not only CTLs, but also DCs and B cells, infiltration and accumulation [114], which implied the formation of TLSs allowing local generation and expansion of tumor-destroying CTLs [84]. In human HCC, CD8+ T cells are usually presented at the peritumoral, rather than intra-tumoral, areas [115]. The combination of the anti-VEGF-A antibody bevacizumab with the anti-PD-L1 antibody atezolizumab showed an unexpectedly high overall response rate, prolonged patient survival, and became a potential first-line treatment option for HCC [116]. Similar treatment success has been seen in metastatic renal cell carcinoma (RCC) [117].

After extravasation from tumor vessels, CTLs are likely to infiltrate into the tumor parenchyma in most malignancies. However, they can also be retained in the tumor margins because some solid tumors, such as PDAC, can establish another physical barrier, the robust stroma. Disruption of the massy stroma might promote CTL penetration and facilitate the antitumor immune response. Focal adhesion kinase (FAK) was identified as a significant contributor to the fibrotic TME and correlated negatively with CD8+ CTL infiltration in human and murine PDACs. By reducing the fibrotic stroma and subsequently enhancing CTL entry, FAK inhibition not only slowed tumor progression but also rendered the previously unresponsive rodent PDAC responsive to PD-1 antagonists [118]. However, whether the additional FAK inhibitor improves tumor sensitivity to ICBs in human PDAC remains elusive. Although FAK inhibitors have been proven as safe and promising [119], to date, few clinical studies have investigated the antitumor effect of combined FAK inhibitors and ICBs in human cancers.

### Chemotherapeutic and targeted agents restrain immunosuppressive cells

The depletion of immunosuppressive cells is involved in the antitumor effect of several agents. Gemcitabine, a nucleoside analog that is commonly used to treat PDAC, depletes circulating, or intra-tumoral MDSCs in multiple cancers. Such depletion favors the restoration of CTL infiltration and cytotoxic activity in both rodent and human cancers [85].

Cyclophosphamide is a nitrogen mustard derivative that is activated intracellularly by phosphoramides or phosphatase, becoming cytotoxic. Tregs were considered to be susceptible to the toxic effects of cyclophosphamide, likely because of their low levels of intracellular antidotes, like glutathione, and their lack of ATP-binding cassette transports, which help to exclude the active metabolite of cyclophosphamide [120, 121]. A low dose of cyclophosphamide has been noted to not only decrease the number but also inhibited the function, of Tregs in rodent tumors [122]. A recent study found that cyclophosphamide preferentially targeted CCR2+ Tregs in a highly active and proliferating state, i.e., the effector Tregs [48]. In humans, metronomic (a repetitive low dose administration) cyclophosphamide treatment effectively reduced both peripheral naïve and activated Tregs, thereby favoring effector T cell subsets in patients with mesothelioma [123]. A clinical trial has also demonstrated that in patients with end-stage metastatic CRC, repetitive low doses of cyclophosphamide induced Treg-deletion and boosted antitumor immunity, which eventually contributed to prolonged progressive-free survival [49]. Similar to cyclophosphamide, camptothecin, a topoisomerase I inhibitor, can also restrain the generation and function of Tregs. It inactivated the transcriptional activity of the NR4A (nuclear receptor subfamily 4 group A) family of nuclear orphan receptors, which inhibited the expression of Forkhead box P3 (FOXP3) and eventually reduced Tregs generation. By removing the suppression by Tregs, irinotecan, a prodrug of camptothecin, promoted the priming and proliferation of CD8+ T cells in the draining lymph nodes and suppressed the growth of murine lung and colon cancer in a CD8+ T cell-dependent manner [50]. Similarly, the chemotherapeutic regimen containing irinotecan, FOLFIRI, was reported to decrease the suppressive activity of peripheral Tregs in patients with CRC [56].

Multi-targeted TKIs, such as sunitinib, inhibit the downstream signaling of receptors, including VEGFR, platelet-derived growth factor receptor (PDGFR), stem cell factor receptor (c-Kit), and colony-stimulating factor -1 (CSF-1) receptor, preventing neoplastic proliferation and tumor angiogenesis [124]. However, these tumor-promoting pathways also play a crucial role in the generation of MDSCs and Tregs. For example, c-Kit receptor signaling and the VEGFA-VEGFR2 pathway are required to generate MDSCs and Tregs [125, 126]. The administration of sunitinib significantly diminished the levels of circulating and intra-tumoral MDSCs, thereby expanding the number of activated tumor-specific CD8+ T cells in murine tumors [127]. Such a relief of MDSC-mediated immunosuppression was also observed in patients with RCC or other metastatic diseases [80, 128]. Similar to MDSCs, Tregs are vulnerable to sunitinib. Recent observations of rodent HCC showed that sunitinib significantly reduced the frequency and function of tumor-infiltrating Tregs, which recovered the cytotoxicity of tumor-specific CD8+ T cells [81]. A combination of sunitinib and anti-PD1 antibodies powerfully activated the antitumor immune response and suppressed tumor growth [82].

The plasticity of macrophages provides an alternative approach to recover antitumor immunity. This comprises repolarizing M2-TAMs toward the pro-inflammatory state (M1 phenotype) in which they act as APCs that facilitate the antitumor immune response. Such a functional transformation has been observed in human PDAC after GEM-based neoadjuvant chemotherapy [55]. Similarly, paclitaxel, one of the most effective cytotoxic agents, which is considered as the standard of care for breast cancer and ovarian cancer, can also repolarize M2-TAMs. Whereas previous studies have shown that TAMs were recruited by cancer cells after paclitaxel treatment and blocked the CD8+ T cell-dependent chemotherapy response [129], paclitaxel was newly identified as an agonist of TLR4 on TAMs and directly polarized this anti-inflammation population into a pro-inflammatory phenotype [59, 60]. The upregulated antigen-presenting ability in this phenotype reversion released CTL-dependent tumor regression [59]. Along this line, patients with breast cancer treated with paclitaxel showed a peripheral pro-inflammatory profile [130]. In addition, an enrichment of genes linked to the inflammatory macrophage phenotype in the TME was reported in patients with ovarian cancer after paclitaxel treatment [59]. Furthermore, TAM repolarization by paclitaxel provides a rationale for combination therapy with ICBs in the treatment of TNBC, in which a high infiltration of immunosuppressive TAMs is associated with a lower response to ICBs [131]. As expected, the combination of atezolizumab and nab-paclitaxel prolonged progression-free survival of patients with metastatic TNBC [132].

## Combination therapies

Numerous clinical trials have been carried out to investigate the combination therapy of immune-modulatory agents and ICBs. An overview of the key studies with their reported results is presented in Table 2.

**Table 2:** Key clinical combination trials

Immune checkpoint blockade	Anticancer agents	Tumor types	Regimen	Result	Reference
Atezolizumab	Carboplatin and etoposide	Extensive-stage NSCLC	Induction: carboplatin AUC 5 + etoposide 100 mg/m^2^ Day 1-3 + atezolizumab or placebo 1200 mg Q3W for 4 cycles; Maintenance: atezolizumab or placebo 1200 mg Q3W	The atezolizumab and chemotherapy combination resulted in significantly longer OS and PFS and became the first-line option for extensive-stage NSCLC.	Horn et al [133]
	Carboplatin and nab-paclitaxel	Stage IV squamous NSCLC	Induction: carboplatin AUC = 6 Day 1 + nab-paclitaxel 100 mg/m2 Day 1, 8 and 15 ± atezolizumab 1200 mg Day 1; Q3W for 4 or 6 cycles; Maintenance: atezolizumab 1200 mg Q3W in the triplet therapy group	The combination of atezolizumab and platinum-based chemotherapy significantly improved PFS in patients with squamous NSCLC; OS was similar between arms.	Jotte et al [134]
	Carboplatin and nab-paclitaxel	Metastatic non-squamous NSCLC	Induction: carboplatin AUC = 6 Day 1 + nab-paclitaxel 100 mg/m2 Day 1, 8 and 15 ± atezolizumab 1200 mg Day1; Q3W for 4 or 6 cycles; Maintenance: atezolizumab 1200 mg Q3W in the triplet therapy group	The combination of atezolizumab and platinum-based chemotherapy significantly improved PFS and OS in patients with metastatic non-squamous NSCLC.	West et al [135]
	Carboplatin, paclitaxel, bevacizumab	Metastatic non-squamous NSCLC	Induction: carboplatin AUC 6 + paclitaxel 200 mg/m^2^ + bevacizumab 15 mg/kg ± atezolizumab 1200 mg, Q3W for 4 or 6 cycles; Maintenance: bevacizumab 15 mg/kg ± atezolizumab 1200 mg, Q3W	The atezolizumab, bevacizumab, and chemotherapy combination significantly improved PFS and OS among patients with metastatic non-squamous NSCLC	Socinski et al [136]
	Nab- paclitaxel	untreated metastatic TNBC	Nab- paclitaxel 100 mg/m^2^ Day 1, 8 and 15 of every 28-day cycle + atazolizumab or placebo 840 mg Q2W	Atezolizumab plus nab-paclitaxel prolonged PFS among patients with metastatic TNBC, especially those with PD-L1 positive tumors.	Schmid et al [132]
		recurrent or metastatic TNBC	Nab- paclitaxel 125 mg/m^2^ day 1, 8 and 15 of every 28-day cycle + atazolizumab 800 mg Q2W	The combination therapy increased antitumor activity (ORR and PFS) and showed manageable toxicity.	Adams et al [137]
	Bevacizumab, sunitinib	Untreated metastatic RCC	Atezolizumab 1200 mg + bevacizumab 15 mg/kg Q3W *vs*. sunitinib monotherapy 50 mg QD for 4 weeks on, 2 weeks off	Atezolizumab plus bevacizumab prolonged PFS versus sunitinib in patients with metastatic RCC (median PFS: 11.2 *vs*. 8.4 months) and showed a favorable safety profile.	Rini et al [117]
	Bevacizumab, sorafenib	Unresectable hepatocellular carcinoma	Atezolizumab 1200 mg + bevacizumab 15 mg/kg day 1 Q3W *vs*. sorafenib 400 mg twice per day Q3W	Atezolizumab plus bevacizumab resulted in better OS and PFS than sorafenib in patients with unresectable hepatocellular carcinoma	Finn et al [116]
	Vemurafenib and cobimetinib	BRAF^V600^- mutated metastatic melanoma	Run-in period (28 days): vemurafenib 960 mg/d BID for 21 days, then 720 mg/d BID for 7 days + cobimetinib 60 mg QD, 1-21 days; Combination period: atezolizumab 800 mg Q2W + vemurafenib 720 mg/d BID and cobimetinib 60 mg QD 1–21 days in 28 days cycle	The triple combination therapy demonstrated promising PFS. The run-in period of vemurafenib and cobimetinib might result in better tolerance and the antitumor response of atezolizumab.	Sullivan et al [138]
		BRAF^V600^- mutated Unresectable locally advanced or metastatic melanoma	Run-In Period (28 days): vemurafenib 960 mg/d BID + cobimetinib 60 mg QD on Days 1 to 21 followed by vemurafenib 720 mg/d BID on Days 22 to 28; Combination Period (Cycle 1 onwards): atezolizumab or placebo 840 mg Day 1 and 15 + cobimetinib 60 mg QD on Days 1 to 21 + vemurafenib 720 mg/d BID on Days 1 to 28 of each 28-day cycle.	The triple combination therapy demonstrated promising PFS *vs*. dual vemurafenib and cobimetinib (median PFS: 15.1 *vs*. 10.6 months). Severe treatment-related adverse events were comparable between the two groups (33.5% *vs*. 28.8%).	McArthur et al [139]
Avelumab	Axitinib or sunitinib	Advanced RCC	Avelumab 10 mg/kg Q2W + axitinib 5 mg BID *vs*. sunitinib monotherapy 50 mg QD for 4 weeks on, 2 weeks off	Avelumab plus axitinib prolonged PFS *versus* sunitinib in patients with advanced RCC (median PFS: 13.8 *vs*. 8.4 months). Grade ≥ 3 treatment-related adverse events were comparable between the two groups.	Motzer et al [10]
Camrelizumab	Decitabine	Relapsed or refractory classic Hodgkin Lymphoma	Camrelizumab 200 mg monotherapy Q3W or decitabine 10 mg/d, days 1 to 5 plus camrelizumab 200 mg, day 8 Q3W	The addition of decitabine to camrelizumab significantly improved the tumor response in patients who were clinically naïve to the PD-1 blockade.	Nie et al [140]
	Gemcitabine and cisplatin	Recurrent or metastatic nasopharyngeal carcinoma	Camrelizumab 200 mg (day 1), gemcitabine 1 g/m^2^ (days 1 and 8), and cisplatin 80 mg/m^2^ (day 1) every 3 weeks followed by camrelizumab 200 mg maintenance once every 3 weeks	The combination of camrelizumab plus gemcitabine and cisplatin has a manageable toxicity profile and promising preliminary antitumor activity in treatment-naive patients.	Fang et al [141]
Durvalumab	Platinum and etoposide	Extensive-stage SCLC	Etoposide 80–100 mg/m^2^ on days 1 to 3 + carboplatin AUC=5/6 or 75–80 mg/m^2^ + durvalumab 1500 mg, Q3W for 4 cycles + maintenance durvalumab 1500 mg Q4W vs. platinum and etoposide for 6 cycles	Durvalumab plus platinum-etoposide significantly improved OS in patients with ES-SCLC *vs*. chemotherapy alone (median OS: 13.0 *vs*. 10.3 months). The safety of the two regimens was similar.	Paz-Ares et al [142]
Ipilimumab	Carboplatin and etoposide	Extensive-stage SCLC	Carboplatin AUC=6 + etoposide 120 mg/m^2^ day 1 and 100 mg day 2 and 3, Q3W up to 6 cycles + ipilimumab 10 mg/kg day 1 of chemotherapy cycles 3-6 and then once every 12-weeks from week 30	The combination therapy showed a beneficial effect in extensive-stage SCLC; however, the toxicity was also significant. Sequential immunotherapy after chemotherapy might be a more feasible approach.	Arriola et al [143]
	Platinum and etoposide	Extensive-stage SCLC	Induction: etoposide 100 mg/m^2^ on days 1 to 3 + carboplatin AUC=5 or cisplatin 75 mg/m^2^ day 1 Q3W for 4 cycles + 4 cycles of ipilimumab or placebo 10 mg/kg Q3W from cycle 3 of chemotherapy; Maintenance: ipilimumab or placebo 10 mg/kg Q12W	The combination of ipilimumab and chemotherapy did not prolong the OS of patients with extensive-stage SCLC.	Reck et al [144]
	Paclitaxel and carboplatin	extensive-disease SCLC	Induction (Q3W for a maximum of 18 weeks): carboplatin AUC=6 + paclitaxel 175 mg/m^2^ vs. concurrent ipilimumab (4 cycles of ipilimumab 10 mg/kg + paclitaxel + carboplatin followed by 2 cycles of placebo + paclitaxel + carboplatin) vs. phased ipilimumab (4 cycles of placebo + paclitaxel + carboplatin followed by 2 cycles of ipilimumab + paclitaxel + carboplatin); Maintenance: ipilimumab for phased- and concurrent-ipilimumab arms) or placebo (control arm) Q12W	Phased ipilimumab, but not concurrent ipilimumab, significantly prolonged immune-related PFS *vs*. chemotherapy alone. A numerical, but not significant, improvement of OS was also observed.	Reck et al [145]
		Advanced squamous NSCLC	Induction: carboplatin AUC = 6 + paclitaxel 175 mg/m^2^ Q3W for 6 cycles + 4 doses of ipilimumab or placebo 10 mg/kg started at cycle 3 of chemotherapy; Maintenance: ipilimumab or placebo once every 12 weeks	The combination of ipilimumab, paclitaxel, and carboplatin did not prolong the OS of patients with advanced squamous NSCLC *vs*. chemotherapy alone.	Govindan et al [146]
Nivolumab	Cisplatin and gemcitabine or pemetrexed; paclitaxel and carboplatin	Advanced NSCLC	Nivolumab 10 mg/kg plus gemcitabine-cisplatin (squamous) or pemetrexed-cisplatin (nonsquamous) or nivolumab 5 or 10 mg/kg plus paclitaxel-carboplatin (all histologies) Q3W for 4 cycles, followed by nivolumab monotherapy every 3 weeks	The combination regimen, especially the paclitaxel-carboplatin plus nivolumab 5 mg/kg, showed encouraging activity (2-year OS rate: 62%). However, the treatment-related adverse events led to greater treatment discontinuation in combination therapies.	Rizvi et al [147]
	Erlotinib	Advanced EGFR-mutant NSCLC	Nivolumab 3 mg/kg every 2 weeks and erlotinib 150 mg/d	The concomitant nivolumab and erlotinib was tolerable and resulted in durable responses in patients with EGFR-mutant, TKI-treated NSCLC.	Gettinger et al [148]
	Cizotinib	ALK-positive NSCLC.	Nivolumab 240 mg every 2 weeks and crizotinib 250 mg twice daily	Such a concomitant regimen of nivolumab and crizotinib resulted in severe, even fatal, hepatic toxicities.	Spigel et al [149]
	Oxaliplatin and S-1 or capecitabine	advanced gastric/gastroesophageal junction cancer	Nivolumab 360 mg day 1+ oxaliplatin 130 mg/m^2^ day 1 + S-1 40 mg/m^2^ or capecitabine 1000 mg/m^2^ twice daily for 14 days followed by 7 days off, Q3W	Nivolumab combined with chemotherapy was well tolerated and demonstrated a higher objective response rate and longer PFS.	Boku et al [150]
	Sunitinib or pazopanib	Advanced or metastatic RCC	Sunitinib (50 mg/day, 4 weeks on/2 weeks off) or pazopanib (800 mg/day) + nivolumab starting dose was 2 mg/kg every 3 weeks, with planned escalation to 5 mg/kg every 3 weeks	The combination therapy resulted in a high incidence of high-grade toxicities (grade 3/4 treatment-related adverse events: 70% – 82%).	Amin et al [151]
Pembrolizumab	Carboplatin and pemetrexed	Non-squamous NSCLC	Carboplatin AUC 5 and pemetrexed 500 mg/m^2^ Q3W for 4 cycles optional pemetrexed 500 mg/m^2^ ± pembrolizumab 200 mg Q3W for 2 years	The triplet therapy could be an effective and tolerable first-line treatment option for patients with advanced non-squamous NSCLC	Langer et al [152]
	Pemetrexed and platinum	Non-squamous NSCLC	Pemetrexed 500 mg/m^2^ + cisplatin 75 mg/m^2^ or carboplatin AUC=5 plus pembrolizumab or placebo 200 mg for 4 cycles, followed by pemetrexed + pembrolizumab or placebo for 35 cycles	The triplet therapy resulted in significantly longer survival (1-year OS rates: 69.2% *vs*. 49.4%, median PFS: 8.8 *vs*. 4.9 months).	Gandhi et al [7]
	Carboplatin and paclitaxel	Squamous NSCLC	Pembrolizumab or placebo 200 mg Q3W for up to 35 cycles + carboplatin AUC6 Q3W and either paclitaxel 200 mg/m^2^ Q3W or (nab)-paclitaxel at 100 mg/m^2^ QW for the first four cycles	The combination therapy resulted in a longer median OS (15.9 *vs*. 11.3 months) and PFS (6.4 *vs*. 4.8 months). This regimen became the first-line treatment.	Paz-Ares et al [8]
	Cyclophosphamide	Sarcoma	Cyclophosphamide 50 mg BID (1 week on and 1 week off), and pembrolizumab 200 mg Q3W	Limited antitumor efficacy might be caused by an immunosuppressive TME.	Toulmonde et al [153]
	Paclitaxel, carboplatin, doxorubicin or epirubicin, and cyclophosphamide	TNBC	Pembrolizumab or placebo 200 mg Q3W + paclitaxel 80 mg/m^2^ QW + carboplatin (QW or Q3W) for 4 cycles, followed by (doxorubicin 60 mg/m^2^ or epirubicin 90 mg/m^2^) + cyclophosphamide 600 mg/m^2^ Q3W + pembrolizumab or placebo 200 mg Q3W for 4 cycles before surgery; followed by 9 cycles of pembrolizumab or placebo 200 mg Q3W post-surgery	The neoadjuvant pembrolizumab - chemotherapy treatment resulted in a significantly higher pathological complete response (64.8% *vs*. 51.2%).	Schmid et al [154]
	5-fluorouracil and cisplatin or carboplatin	HNSCC	Pembrolizumab 200 mg Q3W up for 35 cycles, carboplatin AUC=5 or cisplatin 100 mg/m^2^ + 5-fluorouracil 1000 mg/m^2^ per day for 4 consecutive days, Q3W for 6 cycles	The triple-therapy was recommended as an appropriate first-line treatment for recurrent or metastatic head and neck squamous cancer.	Barbara et al [155]
	Axitinib	RCC	Axitinib 5 mg BID and pembrolizumab 200 mg Q3W	The treatment combination led to significantly longer survival (1-year OS rates: 89.9% *vs*. 78.3%, median PFS: 15.1 vs. 11.1 months) as well as a higher objective response rate (59.3% *vs*. 35.7%).	Atkins et al [156] Rini et al [157]
	Dabrafenib and trametinib	*BRAF*^V600^-mutated melanoma	Concomitant dabrafenib 150 mg/day in divided dose (BID) + trametinib 2 mg QD + pembrolizumab 2 mg/kg Q3W up to 2 years	The triple-therapy was feasible for patients with BRAF^V600^-mutated melanoma, especially those with poor prognostic factors. However, it also significantly increased the grade ≥ 3 treatment-related adverse events.	Ribas et al [110] Ascierto et al [111]
Toripalimab	Axitinib	Metastatic mucosal melanoma	Toripalimab 1 or 3 mg/kg Q2W + axitinib 5 mg BID	The combination of toripalimab plus axitinib was tolerable and showed promising antitumor activity (ORR 48.3%).	Sheng et al [158]

ALK: ALK receptor tyrosine kinase; AUC: area under the curve; BID: twice daily; BRAF: B-Raf proto-oncogene, serine/threonine kinase; EGFR: epidermal growth factor receptor; NSCLC: non-small cell lung cancer; HCC: hepatocellular carcinoma; HNSCC: head and neck squamous cancer; ORR: objective response rate; OS: overall survival; PFS: progression-free survival; QW/Q2W/Q3W: every 1/2/3 weeks; QD: once daily; RCC: renal cell cancer; SCLC: small-cell lung cancer; TME: tumor microenvironment; TNBC: triple-negative breast cancer

Currently, the majority of combination therapies comprise adding concurrent ICBs to existing chemotherapy or targeted regimens. The chemo-immunotherapy usually consists of several cycles of induction concurrent therapy and subsequent maintenance ICB monotherapy, while targeted-immunotherapy is a continuous concurrent regimen. In most combinations, compounds are given in a full dose. The synergistic antitumor effects of these combination therapies have been demonstrated in various cancers, as mentioned before. Several regimens have been suggested as new first-line treatments [133, 152, 155].

Although encouraging progress has been made, the therapeutic efficacy of the current combination therapy remains unsatisfactory. Besides, increased toxicity is another critical issue that should not be overlooked. The incidence of high-grade treatment-related adverse effects (TRAEs) resulting from combination therapy is usually higher than 50%. Unacceptable hepatic toxicity has been seen in the combination of ipilimumab and the BRAF inhibitor vemurafenib during the treatment of melanoma and has resulted in the interruption of this trial [159]. A similar failure happened in the combination of nivolumab and crizotinib in NSCLC [149]. Furthermore, even though in most cases, TRAEs can be managed through the reduction or interruption of drugs, this impairs therapeutic efficacy [146].

Few trials have investigated how the sequence of administration affects the benefit. However, preclinical studies have demonstrated the relationship. Pretreatment with the MEK inhibitor selumetinib significantly augmented the antitumor efficacy of subsequent anti-CTLA4 monotherapy, while the concurrent regimen did not [71]. Similarly, cyclophosphamide administered 1 day before could enhance the antitumor effect of anti-CTLA4 antibody, whereas, treatment with the reversed-sequence regimen led to the apoptosis of proliferating tumor-specific CD8+ T cells and then attenuated tumor control [160]. These observations indicated that induction of chemotherapy or targeted therapy might optimize the TME, thereby supporting the efficacy of subsequent ICBs, at least in ipilimumab-based combination therapies. A previous clinical trial demonstrated that a phased regimen (inductive chemotherapy alone, before concurrent ipilimumab) but not a concurrent regimen (initiating concurrent chemo-ipilimumab) improved the PFS of patients with extensive-disease SCLC [145]. Another regimen comprising phased ipilimumab started at the third cycles of carboplatin and etoposide also showed benefit [143].

The dose of compounds is another important factor for combination therapy. Continuous full dose dual MAPK inhibitors might be inappropriate for combination therapy with ICBs because the increment in intra-tumoral T cells mediated by the inhibitors occurs in the early phase after treatment initiation and become less frequent beyond 2 weeks [161]. Besides, the toxicity of concomitant full-dose of triplet BRAF, MEK, and anti-PD1/PD-L1 inhibitors significantly limited their efficacy [110, 111]. A phase II study has investigated different doses of BRAF and MEK inhibitors in combination therapy with pembrolizumab in BRAF^V600^-mutant melanoma. Lower rates of high-grade TRAEs and higher objective response rates (ORR) were found in patients treated with short-term intermittent dual MAPK inhibitions, rather than pembrolizumab plus continuous targeted treatment [162]. However, because the high ORR did not mean a better survival outcome in all cases, whether the intermittent regimen would be the more efficient and safe approach should be tested in a larger cohort and with longer follow-up. However, these findings did suggest exploring the optimal dose in future combination trials.

## Perspective and conclusion

ICB-based cancer immunotherapy removes the checkpoint constraints on adaptive antitumor immunity, thereby releasing the cytotoxicity of tumor-specific CD8+ T cells. It can induce tumor shrinkage, durable disease control, and prolonged survival, but only in a minority of patients, likely because the CD8+ T cell-mediated antitumor immune response is impaired in most cancers. Antitumor immunity is mainly driven or suppressed by cellular factors in the TME; therefore, treatment strategies that can specifically modify the TME toward an inflamed phenotype are expected to be combined with ICBs to augment their therapeutic efficacy.

The therapeutic effects of chemotherapy and targeted therapy are traditionally considered to rely on tumor cell-intrinsic sensitivity or cancer-specific alterations. However, the empirical selection of clinically efficient therapeutic regimens might also imply enhanced anticancer immunosurveillance. For example, anthracyclines-based regimens are commonly used to treat breast cancers which are usually considered as nonimmunogenic with rare CD8+ T cell infiltration. Gemcitabine is the frontline treatment option for PDAC, which is characterized by abundant MDSCs and TAMs. These “coincidences” suggest that the TME or cancer-immunity cycles might insensibly affect the selections of standard chemotherapy. Furthermore, it indicates that chemotherapy and targeted therapy are promising candidates to sensitize tumors to ICB therapy because they can target the impaired steps of the cancer-immunity cycle in certain tumors. Indeed, most of the current combination regimens comprise the concurrent administration of ICBs and the existing chemotherapeutic regimen and are currently the best choice to improve patient survival.

Optimization of the drug combination, dose, and sequence is still needed to achieve maximum therapeutic efficacy. Although the existing clinically efficient chemotherapeutic regimens might have specifically enhanced anticancer immunosurveillance for each cancer, whether these empirical regimens are optimal for combination therapy with ICBs remains unknown. A comprehensive understanding of the composition and immune situation of the TME and a careful examination of the immunological characteristics of the currently used chemotherapeutics would be conducive to selecting the most ideal drugs for combination therapy of cancer. In addition to drug combinations, the dose and sequence of administration of chemotherapeutics and ICBs can also affect the therapeutic effect. The long-term and full-dose administration of chemotherapeutics might be unnecessary in the combination regimen because it not only results in more serious toxicity but also damages, rather than enhances, anti-tumor immunity. These findings suggest that there is a need to investigate the optimal doses of chemotherapeutics in combination therapy. Besides, chemotherapy induction before ICB administration seems to be beneficial. In such induction phases, chemotherapeutics promote the generation and infiltration of CTLs or delete the immunosuppressive cells, thereby optimizing the TME for subsequent ICB therapy. Serial histological examination during chemotherapy can reveal the dynamic changes of the cancer immune context, thus favoring the selection of the best combination treatment time and sequence.

Similar to chemotherapeutics, radiotherapy has profound immunomodulatory effects. Through inducing DNA damage, radiotherapy can expand the spectrum of neoantigens and upregulate the antigen-presenting machinery in tumor cells [163]. Besides, radiation has been proved as an ICD-inducer and can promote the recruitment of DCs and CTLs through DAMPs and chemokines [164]. However, the radiotherapy-mediated immuno-stimulations are usually blunted by the cancer cell-intrinsic DNA damage response (DDR) and immunosuppressive cells, including MDSCs and TAMs. DDR inhibitors have been shown to further potentialize the radiation-induced inflammation in TME [165]. Furthermore, as chemotherapeutics like paclitaxel can deplete or convert the suppressor cells, the combinations of radiotherapy with chemo-/targeted therapy might further enhance the antitumor immune response and ICB therapy. However, few studies have investigated whether the triplet of chemo-radio-immunotherapy actually works.

In summary, the immunomodulatory effect of chemotherapeutics provides a strong cancer biology rationale for their combination with ICBs. Such combinations will not only directly inhibit malignant cells but also augment the immune recognition and elimination of tumor cells. Furthermore, it establishes long-term antitumor memory and thus might represent a curative treatment. The synergistic antitumor efficacy of combination therapy has been demonstrated in various cancers; however, the maximum benefit has not yet been achieved. Future studies that evaluate the therapeutic efficacy of regimens with different drug combinations, doses, and sequences will help to develop the optimal combination therapy for each cancer.

## Data Availability

Not applicable.

## Abbreviations

ALK: Anaplastic lymphoma kinase
ANGPT2: Angiopoietin-2
APC: Antigen-presenting cells
ATP: Adenosine-triphosphate
AUC: Area under the curve
BID: Twice daily
BRAF: B-Raf proto-oncogene, serine/threonine kinase
BRCA: Breast cancer susceptibility gene
Breg: Regulatory B cell
CCR2: Chemokine (C-C motif) receptor 2
cGAS-STING: Cyclic GMP–AMP synthase/stimulators of interferon gene
CRC: Colorectal cancer
CSF-1: Colony-stimulating factor -1
CTL: Cytotoxic T lymphocytes
CTLA-4: Cytotoxic T lymphocyte-associated protein 4
CXCL9/10/11: C-X-C motif chemokine ligand 9/10/11
CXCR3: C-X-C motif chemokine receptor 3
DAMP: Damage-associated molecular pattern
DC: Dendritic cells
DDR: DNA damage response
EGFR: Epidermal growth factor receptor
ERK: Extracellular regulated kinase
FAK: Focal adhesion kinase
FOLFIRI: Eolinic acid, 5-fluorouracil
FOXP3: Forkhead Box P3
γ/δ T cell: Gamma/delta T cell
GSDM: Gasdermin
HCC: Hepatocellular carcinoma
HMGB1: High-mobility group box1
HNSCC: Head and neck squamous cell carcinoma
ICAM-1: Intercellular adhesion molecule–1
ICB: Immune checkpoint blockade
ICD: Immunogenic cell death
IFN-γ: Interferon-gamma
MDSC: Myeloid-derived suppressor cell
NK cells: Natural killer cells
NR4A: Nuclear receptor subfamily 4 group A
NSCLC: Non-small cell lung cancer
ORR: Objective response rate
OS: Overall survival
PARPi: Poly (ADP-ribose) polymerase inhibitor
PD-1: Programmed cell death 1
PDAC: Pancreatic ductal adenocarcinoma
PDGFR: Platelet-derived growth factor receptor
PD-L1: Programmed death-ligand 1
PFS: Progression-free survival
PGE2: Prostaglandin E2
QD: Once daily
QW/Q2W/Q3W: Every 1/2/3 weeks
RCC: Renal cell carcinoma
ROS1: ROS proto-oncogene 1, receptor tyrosine kinase
SCLC: Small cell lung cancer
TAM: Tumor-associated macrophage
TAN: Tumor-associated neutrophils
TCR: T-cell receptor
TGF-β: Transforming growth factor-beta
TKI: Tyrosine kinase inhibitor
TLR: Toll-like receptor
TLS: Tertiary lymphoid structures
TMB: Tumor mutation burden
TME: Tumor microenvironment
TNBC: Triple-negative breast cancer
TRAE: Treatment-related adverse effect
Treg: Regulatory T cell
VCAM-1: Vascular cell adhesion molecule–1
VEGF-A: Vascular endothelial growth factor A
